# The Uniform System of Accounts

**Published:** 1920-02-28

**Authors:** H. E. Smithers

**Affiliations:** General Manager, The Scientific Press, Limited. 28 and 29 Southampton Street, W.C.


### HOSPITAL ACCOUNTS.

*To the Editor of* The Times.

Sir,—The letter from Mr. J. Danvers Power as it appears in *The Times* of the 17th inst. does not contain all the facts, and if left as it stands must inevitably cause serious misapprehension. The correct position was stated by the Marquess of Cambridge (then Duke of Teck) in his speech as presiding governor of King Edward's Hospital Fund for London at St. James's Palace on December 12, 1913, as follows :—

"The comparisons of cost per bed depend for their accuracy very largely on the degree of uniformity with which the accounts and statistics of the various hospitals are made up. For this uniformity we are indebted to the uniform system of hospital accounts introduced by Sir Henry Burdett, and to the revision of that system adopted by the three funds—the Sunday Fund, the Saturday Fund, and ourselves—seven years ago. Since then certain minor differences in the method of applying the rules of the system have grown up, and during the year a joint committee of the three funds has issued some supplementary regulations and suggestions. Mr. Griffiths, who was responsible for the original revision, again gave his expert aid as the representative of this fund; and, again, as on the previous occasion, a committee of hospital secretaries assisted with their practical experience."

The letter you publish in *The Times* of the 19th inst., signed by Mr. R. J. Bland, demonstrates that he has not made himself familiar with the facts. His letter exhibits an ignorance of Sir Henry Burdett's system, which is surprising in the case of a hospital secretary. In the second edition of Sir Henry Burdett's Uniform System of Hospital Accounts, published in 1903, a whole chapter, Chapter IV., is devoted to the balance-sheet, and a form of balance-sheet is also given. It is explained that the author

"is aware of instances where the authorities object to inform the public of the fact that the hospital possesses large assets, apart from those which are currently available. We know that such reticence has the effect of misleading the charitable public, and should not be practised. Hence it is that we have drafted the accompanying balance-sheet on the assumption that it must be the wish of the managers of every well-conducted hospital and institution that all the estates and possessions should be set out fully and precisely."

It will be seen, therefore, that Mr. Bland's statement that Sir Henry Burdett's "uniform system consisted only of an income and expenditure account" is devoid of foundation and contrary to the fact. The second edition of Sir Henry Burdett's Uniform System, which includes the balance-sheet, was published in February 1903; while the first edition of the Red-book, the Uniform System of Hospital Accounts, as revised and adopted by the King's Fund and the Hospital Sunday and Saturday Funds, is dated December 1906. Sir Henry Burdett was called into consultation several times in connection with the revision of the Uniform System.—I am, yours faithfully,

H. E. Smithers,
General Manager, The Scientific Press, Limited.
28 and 29 Southampton Street, W.C., February 19.

